# Efficient Recovery of Collagen from Tannery Waste Materials and Its Integration into Functional Hydrogel Systems

**DOI:** 10.3390/gels12040301

**Published:** 2026-04-01

**Authors:** Ilnaz Fargul Chowdhury, Akash Debnath, Shyama Prosad Moulick, Md. Ashraful Alam, S. M. Asaduzzaman Sujan, Md. Tushar Uddin, Md. Salim Khan, Ajoy Kanti Mondal

**Affiliations:** 1 Institute of National Analytical Research and Service, Bangladesh Council of Scientific and Industrial Research, Dhanmondi, Dhaka 1205, Bangladesh; 2 Leather Research Institute, Bangladesh Council of Scientific and Industrial Research, Savar, Dhaka 1350, Bangladesh; 3 BCSIR Dhaka Laboratories, Bangladesh Council of Scientific and Industrial Research, Dhanmondi, Dhaka 1205, Bangladesh; 4 Institute of Energy Research and Development, Bangladesh Council of Scientific and Industrial Research, Dhanmondi, Dhaka 1205, Bangladesh

**Keywords:** raw trimmings, collagen, hydrogel, biomaterial, self-healing, antibacterial

## Abstract

The development of multifunctional, mechanically robust, and sustainable hydrogels from renewable biomaterials has attracted increasing attention for advanced biomedical applications; however, achieving an optimal balance between mechanical stability, biofunctionality, and infection control remains challenging. In this work, collagen (COL) extracted from raw trimming wastes from a tannery is used to fabricate COL/PAA/Fe composite hydrogels via the ammonium persulfate (APS)-initiated polymerization of acrylic acid (AA) coupled with Fe^3+^-mediated coordination cross-linking. The resulting hydrogel network is stabilized by synergistic COL-poly(acrylic acid) (PAA) hydrogen bonding and dynamic Fe^3+^–carboxylate coordination, imparting enhanced mechanical strength and elasticity. The optimized hydrogel exhibited maximum tensile and compressive strengths of ~0.176 MPa at 751% elongation and ~1.945 MPa at a strain of 80%, respectively. In addition, a high ionic conductivity of 4.11 S·m^−1^ is achieved, enabling structural integrity under deformation and suitability for flexible electronic interfaces. The prepared hydrogel also displayed rapid autonomous self-healing behavior and substantial antibacterial properties against both Gram-positive and Gram-negative bacteria. Overall, COL is employed herein as a sustainable precursor, highlighting an eco-conscious approach to biomaterial design. This work presents a versatile strategy for producing mechanically stable and biofunctional hydrogels with strong potential for wound dressing, tissue engineering, and injectable biomedical applications.

## 1. Introduction

The leather industry is a significant contributor to the global economy; however, its manufacturing processes generate a large amount of solid waste, varying in composition and quantity at various stages [1]. In countries like Bangladesh, where millions of bovine and caprine hides are processed annually, the improper management of tannery residues—particularly raw trimmings rich in proteinaceous matter—has intensified concerns related to soil contamination, water pollution, and chemical toxicity [2,3]. Despite this burden, these wastes contain high proportions of structurally valuable biomolecules, most notably collagen (COL), which remains largely underutilized [4]. Transforming this abundant yet overlooked resource into high-value biomaterials represents an important opportunity to support circular bioeconomy goals, reduce ecological footprints, and create sustainable material platforms [5].

COL is a naturally occurring polymer that constitutes the main structural framework of connective tissues such as bones, tendons, and cartilage [6]. Owing to its excellent biocompatibility, low immunogenicity, and wide availability, COL has attracted growing interest as a versatile biomaterial for diverse biomedical applications [7,8,9]. COL-based materials have been extensively employed in clinical applications, for example, vascular grafts, aortic heart valves, wound dressing, and drug delivery matrices [10]. Worldwide surveys indicate that approximately 325,000 tons of trimming wastes are generated annually as by-products of the leather industry, and considering a minimum COL content of 20% in raw trimmings, nearly 65,000 tons of COL is potentially recoverable each year [11]. In Bangladesh alone, an estimated 12,271.35 metric tons of raw trimming waste could be valorized into approximately 2454.27 metric tons of COL annually [12]. Although several approaches have been reported for the utilization of COL protein derived from these residues [5,13], a significant proportion of this valuable biomolecule remains underexploited, highlighting the need for efficient and sustainable strategies to convert tannery-derived COL into high-value functional biomaterials.

Hydrogels are a class of three-dimensional cross-linked network of hydrophilic polymers capable of high water retention and structural integrity, making them well suited for a wide range of functional applications [14,15,16]. Among various hydrogel-forming polymers, COL has emerged as a highly promising candidate, offering hydrated networks that closely resemble the native extracellular matrix while providing tunable mechanical, physicochemical, and biological properties [17]. The fundamental structure of COL is a triple helix composed of three left-handed polypeptide chains [18]. The presence of free functional groups in COL, particularly amine and carboxyl groups, enables structural modification and the formation of physical or chemical cross-links [19]. This inherent ability of COL to establish intra- and inter-fibrillar cross-linking can be strategically exploited to fabricate hydrogels with tunable mechanical properties, allowing them to closely match the mechanical behavior of surrounding tissues upon implantation [20,21]. For instance, Kang et al. developed a COL–hyaluronic acid hydrogel capable of rapid in situ gelation at corneal defect sites, enabling sustained drug release while exhibiting pronounced pro-regenerative and anti-inflammatory effects [22]. However, the use of bioorthogonal cross-linkers in this work may pose challenges, including potential cytotoxicity, immune responses, and increased synthesis complexity, which could affect reproducibility, hydrogel uniformity, and in vivo performance.

Some recent studies have focused on the incorporation of metal ions into hydrogel matrices to improve both their structural integrity and functional performance [23,24,25]. Introducing transition metal ions, such as Fe^3+^, in collagen-based hydrogels offers several distinct advantages, including increased conductivity arising from the presence of mobile metal ions within the hydrated network [26]. Fe^3+^ ions can serve as effective physical cross-linkers, forming stable coordination bonds with the amino acid residues of COL, thereby improving the mechanical strength of the hydrogel network [27]. Moreover, the Fe^3+^/Fe^2+^ redox pair enables the formation of dynamic, reversible redox-mediated interactions, which can impart self-healing and stimuli-responsive behavior to the hydrogel [28]. Beyond mechanical reinforcement, Fe^3+^ ions can also influence polymerization kinetics in the presence of APS, promoting more uniform network formation and hydrogel homogeneity [29]. These combined effects make Fe^3+^-incorporated COL-based hydrogels particularly attractive for biomedical and functional material applications, where enhanced ionic conductivity, tunable mechanics and adaptive responsiveness are highly desirable.

This study reports the development of a COL-based hydrogel synthesized from COL extracted directly from raw trimming waste, combined with acrylic acid (AA), FeCl_3_, and APS. AA was employed as the monomer and rapidly polymerized through an APS-initiated free-radical process to form the hydrogel network without any external stimulus. The incorporation of Fe^3+^ ions introduced dynamic, reversible coordination cross-links and a Fe^3+^/Fe^2+^ redox system, endowing the hydrogel with self-healing properties. To the best of our knowledge, this is the first study to integrate waste-derived COL with a Fe^3+^/APS-mediated polymerization system to produce a mechanically robust hydrogel with self-healing and antibacterial properties. The resulting material not only demonstrates significant potential for wound healing but also paves the way for future research into advanced biomedical applications, including drug delivery, tissue engineering, and regenerative medicine.

## 2. Results and Discussion

### 2.1. Formation Mechanism of the COL/PAA/Fe Hydrogel

**Figure 1:**
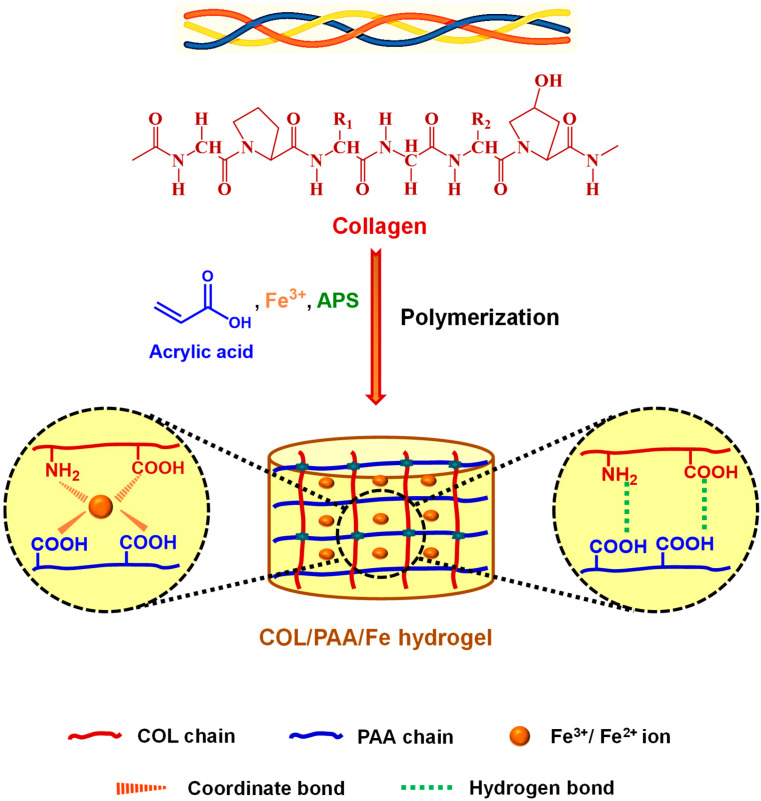
Possible formation mechanism of the COL/PAA/Fe hydrogel (Three different colors of COL structure represent the triple helix structure of COL).

Multifunctional COL/PAA/Fe hydrogels were fabricated in the present study by utilizing COL extracted from raw trimmings as a sustainable and value-added resource. When derived from tannery residues, these hydrogels not only enable the high-value utilization of waste-derived COL but also align with circular bioeconomy principles by reducing environmental burdens associated with leather-processing waste streams. Figure 1 illustrates a possible mechanism underlying hydrogel formation. The presence of AA, Fe^3+^ and APS in the system initiated a rapid polymerization reaction, with APS acting as the radical initiator. Upon mixing and mild heating, rapid gelation occurred, indicating efficient cross-linking and the formation of a homogeneous, self-supporting three-dimensional hydrogel network, as observed macroscopically. In contrast, previous studies have commonly employed acrylamide as the monomer and N, N′-methylenebisacrylamide as the cross-linker for hydrogel synthesis, which typically required prolonged gelation times and elevated external temperatures, along with the addition of separate cross-linking agents [30,31,32]. Notably, in this work, the COL/PAA/Fe hydrogel was synthesized within 2 min without the use of external stimuli or supplementary cross-linkers, highlighting the novelty and ease of the proposed preparation process.

The formation of the hydrogel network may involve multiple, synergistic interactions. Collagen (COL), a natural protein with a triple-helical structure, is rich in functional groups such as amino (–NH_2_), hydroxyl (–OH), and carboxyl (–COOH) moieties, which enable multiple interactions within the hydrogel network [19,33]. APS acts as a free-radical initiator, generating sulfate radicals under mild heating, which initiate the polymerization of AA to form poly(acrylic acid) (PAA) chains [34]. These covalent PAA chains are likely to serve as the primary polymer network, contributing to the structural backbone and elasticity of the prepared COL/PAA/Fe hydrogel.

**Figure 2:**
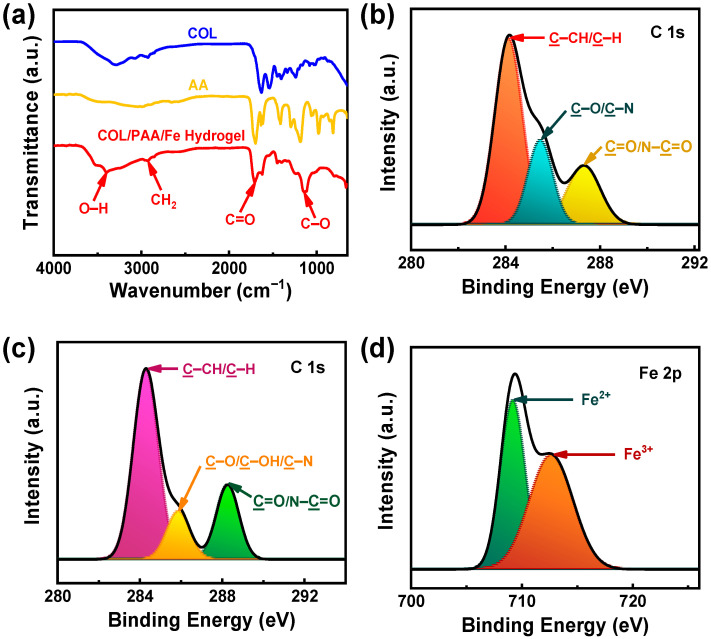
(**a**) FT-IR spectra of COL, AA, and the COL/PAA/Fe hydrogel; C 1s XPS spectrum of (**b**) COL, (**c**) COL/Fe^3+^ chelated suspension, and (**d**) XPS spectrum of Fe 2p of the COL/PAA/Fe hydrogel.

Fe^3+^ ions can act as multivalent cross-linkers, coordinating with the functional groups (–NH_2_ and –COOH) in COL and PAA to form stable metal–ligand complexes (Figure 1), which contribute to the mechanical integrity and high IC of the hydrogel [35]. Evidence for such coordination interactions is supported by the FT-IR results (Figure 2a), where the characteristic carbonyl (C=O) stretching band of AA shows a shift and reduced intensity after hydrogel formation, indicating the participation of carboxylate groups in coordination with Fe^3+^ ions. Additionally, the broad –OH/–NH stretching band exhibits a noticeable shift and broadening, suggesting the formation of new hydrogen bonding interactions between COL and PAA chains during gelation.

The Fe^3+^/Fe^2+^ redox pair may also enable dynamic and reversible redox-mediated interactions, thereby imparting self-healing and stimuli-responsive properties to the hydrogel [28]. XPS analysis further supports this mechanism by revealing shifts in the C 1s binding energies and an increased contribution of C=O/N–C=O components after Fe^3+^ incorporation (Figure 2c), indicating changes in the chemical environment of carbon-containing functional groups due to interactions among COL, PAA, and Fe ions. Moreover, the simultaneous presence of Fe^3+^ and Fe^2+^ signals in the XPS spectrum (Figure 2d) suggests that redox processes occur during hydrogel formation, contributing to dynamic interactions within the network.

Additionally, hydrogen bonding interactions between the –NH_2_ and –OH groups of COL and the –COOH groups of PAA (Figure 1) are likely to provide secondary stabilization, improving network cohesion and elasticity [36]. The FT-IR spectral shifts observed in the amide and C–O stretching regions (Figure 2a) further indicate strong intermolecular interactions between COL and PAA chains.

The combined effect of redox-initiated radical polymerization, metal coordination, and hydrogen bonding resulted in the formation of a robust, multifunctional hydrogel network. The rapid gelation observed visually confirms the efficiency of this synergistic mechanism. Furthermore, the hydrogel exhibits elasticity and shape retention under handling, demonstrating the effectiveness of Fe^3+^ coordination and hydrogen bonding in reinforcing the polymerized PAA-COL matrix.

### 2.2. Structural Characterizations of the Synthesized COL/PAA/Fe Hydrogel

Figure 2a presents the FT-IR spectra of COL, AA, and the COL/PAA/Fe hydrogel, revealing distinct spectral features and notable changes upon hydrogel formation.

The FT-IR spectrum of COL (Figure 2a) exhibits characteristic absorption bands associated with its polypeptide backbone. The broad band observed at 3294 cm^−1^ corresponds to the stretching vibrations of –OH and –NH groups of amide A [37], indicative of extensive hydrogen bonding within COL. Characteristic peaks are found at 3075 cm^−1^ for the stretching vibration of amide B; and at 2929 cm^−1^ for the asymmetrical stretching vibration of −CH_2_− of amide B [38]. Additionally, some prominent peaks for amide I (−C=O stretching vibration), amide II (N−H bending vibration), and amide III (C−N stretching vibration) are found at approximately 1634, 1544, and 1238 cm^−1^, respectively [39], confirming the preservation of the COL triple-helical structure.

The FT-IR spectrum of AA (Figure 2a) shows a strong absorption band at 1705 cm^−1^, attributed to the C=O stretching vibration of the carboxylic acid group. The broad –OH stretching band around 2983 cm^−1^ is characteristic of hydrogen-bonded carboxylic acid dimers, while the bands near 1450–1400 cm^−1^ correspond to C–H bending vibrations of the vinyl group [40].

Upon formation of the COL/PAA/Fe hydrogel, the O-H stretching band exhibited a slight shift to 3392 cm^−1^ and broadening, suggesting the establishment of new hydrogen bonds between COL and PAA chains during gelation (Figure 2a) [36]. Similarly, the symmetric stretching vibration of −CH_2_− in the pyranose ring is shifted from 2929 to 2935 cm^−1^. The peak observed at 1711 cm^−1^, associated with the stretching vibration of carbonyl groups (–C=O), indicates the formation of cross-links within the hydrogel matrix. Notably, the C=O stretching band of AA shifted to lower wavenumbers and decreased in intensity, suggesting the involvement of carboxylate groups in coordination interactions with Fe^3+^ ions and hydrogen bonding with COL functional groups. Moreover, transmittance peaks for amide I and C−O stretching are shifted from 1634 to 1711 cm^−1^ and 1072 cm^−1^ to 1128 cm^−1^, respectively. These can be attributed to asymmetric and symmetric stretching vibrations of coordinated carboxylate (–COO^−^) groups, providing strong evidence of Fe^3+^-mediated ionic crosslinking within the hydrogel network [41]. The reduced intensity and slight broadening of COL amide bands further imply molecular interactions between COL and PAA chains. The disappearance of the vinyl group bands in the hydrogel also confirms the polymerization of AA to PAA.

Therefore, the FT-IR results confirm that the COL/PAA/Fe hydrogel is stabilized through a combination of hydrogen bonding between –NH_2_/–OH groups of COL and –COOH groups of PAA, as well as coordination interactions between Fe^3+^ ions and carboxylate groups, leading to the successful formation of a robust and interconnected hydrogel network.

XPS analysis was performed under ambient conditions. The C 1s XPS spectrum of COL extracted from raw trimmings (Figure 2b) exhibits characteristic peaks at binding energies of 284.1, 285.5, and 287.3 eV, which are attributed to C-CH/C-H, C-O/C-OH/C-N, and C=O/N-C=O bonds, respectively. These findings are consistent with previously reported results for COL [15,42].

In the COL/Fe^3+^ chelated suspension (Figure 2c), the corresponding C 1s peaks appear at 284.3, 285.8, and 288.3 eV, indicating the presence of the same bonds. Minor shifts observed in the C 1s peak positions are likely attributable to interactions among different functional groups in collagen, polyacrylic acid, and Fe^3+^ ions. XPS analysis reveals that the relative intensity of C=O/N-C=O peak in COL/Fe^3+^ chelated suspension is higher compared to the C-O/C-OH/C-N peak, in contrast to COL. This increase may result from the transformation of C-O/C-OH/C-N functionalities in COL into carbonyl groups following Fe^3+^ incorporation [23].

The reducing groups in COL (–OH and –COOH) are prone to oxidation. The introduction of Fe^3+^ into the COL/AA mixture leads to its partial reduction to Fe^2+^, as evidenced by the simultaneous presence of Fe^3+^ and Fe^2+^ signals in the XPS spectrum (Figure 2d). The elevated content of C=O groups observed in the COL/Fe^3+^ chelated suspension (Figure 2c) can also be attributed to the involvement of the Fe^3+^/Fe^2+^ redox couple in the reaction.

Overall, these results provide clear evidence of dynamic redox interactions during hydrogel formation, which play a crucial role in the effective preparation of the COL/PAA/Fe hydrogel.

### 2.3. Process Optimization for Hydrogel Fabrication

**Figure 3:**
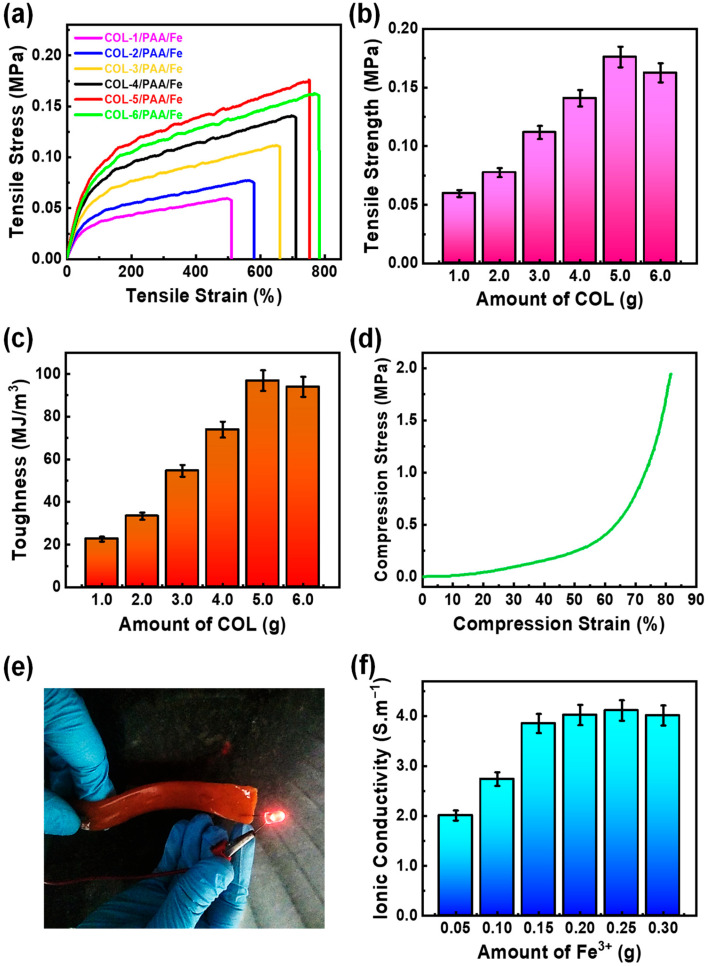
(**a**) Tensile stress–strain plots of different COL/PAA/Fe hydrogels, (**b**) Tensile strengths, and (**c**) Toughness of different COL/PAA/Fe hydrogels, (**d**) Compressive stress–strain curve of the COL-5/PAA/Fe hydrogel, (**e**) LED bulb lights up instantly, showcasing the synthesized hydrogel’s superior ionic conductivity (IC), and (**f**) Influence of Fe^3+^ content on the IC of the COL-5/PAA/Fe hydrogel.

The preparation procedure of the COL/PAA/Fe hydrogels was systematically optimized, with tensile strength measurements initially employed to identify the optimal COL content (Figure 3a). This was achieved by varying the COL content during hydrogel fabrication while keeping all other components constant (Table 1). With increasing amounts of COL in the hydrogel, the tensile strengths of the resulting hydrogels initially increased, reaching a peak, and then decreased at higher concentrations (Figure 3a,b). Highest tensile strength was observed when the hydrogel contained approximately 5.0 g of COL, and this amount was therefore selected for subsequent experiments. At this concentration, the interactions (for example, effective coordination/complexation, cross-linking, and hydrogen bonding) among COL, PAA and Fe^3+^ are likely strongest, thereby giving rise to the maximum tensile strength of the COL/PAA/Fe hydrogel [27].

The synthesized hydrogel possessed excellent ionic conductivity (IC), allowing it to instantly illuminate an LED bulb with high brightness (Figure 3e and Video S1). The effect is largely due to the incorporation of Fe^3+^ ions into the COL/PAA/Fe hydrogel and the abundance of functional groups in COL (–NH_2_, –OH, –COOH), which dissociate in water to facilitate ion transport and enhance conductivity [35]. Dissociation of the –COOH groups in COL can lead to the formation of carboxylate anions (–COO^−^), contributing to the hydrogel’s overall ionic charge and improved conductivity.

The IC of the hydrogel strongly depended on the amount of FeCl_3_·6H_2_O, which was optimized accordingly (Table 2). The conductivity of the COL-5/PAA/Fe hydrogel initially increased as the FeCl_3_·6H_2_O content was raised, attaining its maximum value of 4.11 S·m^−1^ at 0.25 g of FeCl_3_·6H_2_O (Table 2 and Figure 3f). The observed rise in conductivity of the hydrogel at higher Fe^3+^ levels may result from the increased supply of charge carriers provided by the additional ions. This enhancement is likely due to effective coordination between Fe^3+^ ions and the functional groups of COL and PAA, which promotes ion transport.

Beyond 0.25 g of FeCl_3_·6H_2_O, increasing the amount of Fe^3+^ had little effect on the IC, and further addition of Fe^3+^ ions in the system caused a reduction in the hydrogel’s conductivity (Figure 3f). This decline is likely due to the precipitation of Fe^3+^ ions and the presence of unreacted ions on the hydrogel surface, consistent with previously reported studies [25,43].

Therefore, based on the optimization studies, 5.0 g of COL and 0.25 g of FeCl_3_·6H_2_O were selected as the ideal amounts for the COL/PAA/Fe hydrogel preparation.

### 2.4. Mechanical Properties

**Figure 4:**
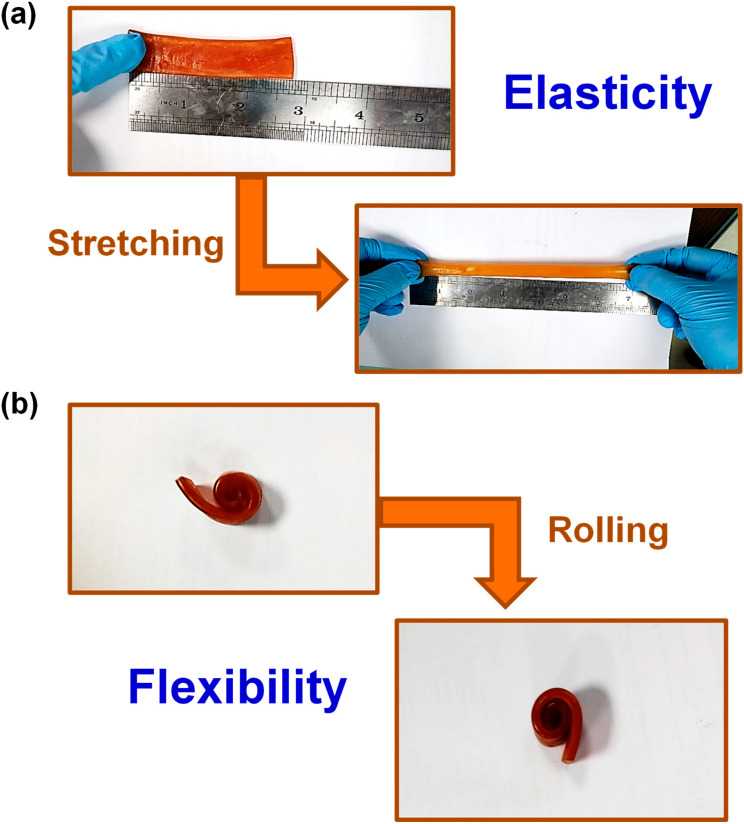
Visual representation of (**a**) Elasticity and (**b**) Flexibility of the COL-5/PAA/Fe hydrogel.

The fabricated COL/PAA/Fe hydrogel exhibited remarkable elasticity and excellent flexibility. To reduce its slight adhesiveness, the hydrogel was allowed to dry naturally at room temperature for 2 days. Once dried, it could be rolled and unrolled multiple times without showing any visible deformation. As shown in Figure 4 and Video S2, the hydrogel demonstrates both high flexibility and elastic recovery. The hydrogels’ capacity for repeated elongation beyond their original lengths highlights the exceptional elastic properties of the synthesized COL/PAA/Fe hydrogels. Maximum stretchability was achieved at about 5.0 g of COL and 0.25 g of FeCl_3_·6H_2_O, where the tensile stress reached ~0.176 MPa at ~751% elongation (Figure 3a). Under these conditions, the hydrogel also exhibited the highest toughness of ~96.89 MJ·m^−3^ (Figure 3c). The compressive strength of the COL-5/PAA/Fe hydrogel was measured to be ~1.945 MPa at a compressive strain of ~80% (Figure 3d).

The outstanding mechanical elasticity and flexibility of the COL/PAA/Fe hydrogels arise from the effective interactions among the various functional groups of COL and PAA, coupled with efficient coordination/complexation of Fe^3+^/Fe^2+^ ions. These interactions enabled efficient intra- and intermolecular cross-linking without the need for additional chemical cross-linkers, resulting in optimized mechanical performance consistent with previous reports [15,23]. The combined effects of redox-initiated radical polymerization, metal–ligand coordination, and hydrogen bonding helped to fabricate a robust and multifunctional hydrogel with tunable mechanical properties.

### 2.5. Self-Healing Performance

**Figure 5:**
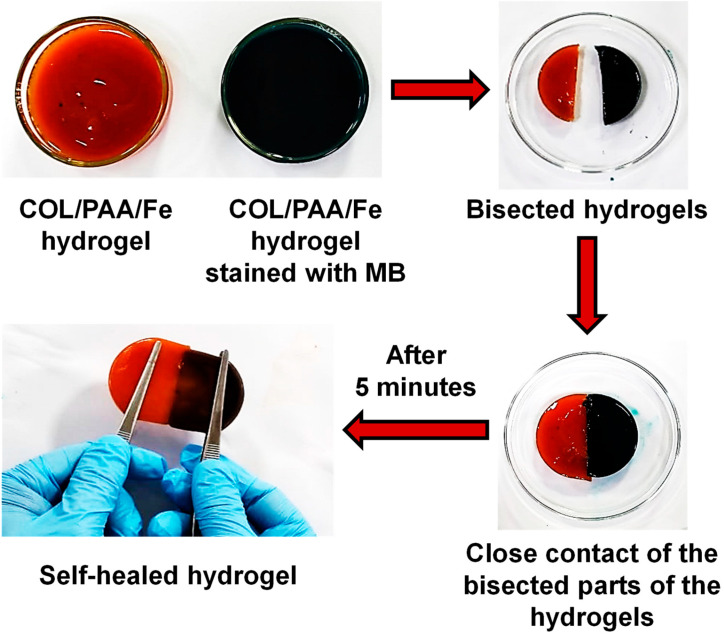
Self-healing performance of the COL/PAA/Fe hydrogel under ambient conditions.

The self-healing capability of the COL/PAA/Fe hydrogel was qualitatively assessed through a visual macroscopic healing assay, as shown in Figure 5. Immediately after being cut, the stained and unstained hydrogel halves were rejoined and allowed to heal autonomously under ambient conditions without external stimulus or applied pressure. After 5 min of contact, the interface between the two halves became indistinguishable, and the hydrogel recovered its structural continuity, indicating rapid and effective self-healing behavior (Figure 5 and Video S3). Additionally, the tensile strength of the self-healed hydrogel was measured (Figure S1). The tensile stress and strain of the self-healed hydrogel were 0.142 MPa and 716% (Figure S1), respectively, which were close to the results of the original hydrogel (0.176 MPa and 751%).

The successful reconnection of the hydrogel pieces demonstrates the presence of dynamic and reversible interactions within the polymer network. In this system, Fe^3+^–carboxylate coordination bonds between Fe^3+^ ions and the –COO^−^ groups of PAA can serve as reversible physical cross-links, enabling bond dissociation and reformation at the damaged interface [28]. Simultaneously, extensive hydrogen bonding between the –NH_2_ and –OH groups of COL and the –COOH groups of PAA is likely to contribute additional dynamic interactions that facilitate network reorganization during healing [44,45]. The APS-initiated polymerization may further facilitate polymer chain diffusion and interpenetration of PAA chains within the COL matrix, enabling rapid structural recovery under ambient conditions [46].

Therefore, the macroscopic self-healing behavior confirms that the COL/PAA/Fe hydrogel possesses an intrinsic, stimulus-free healing mechanism driven by synergistic metal–ligand coordination and hydrogen bonding interactions. This dynamic network architecture endows the hydrogel with enhanced durability and resilience, making it particularly attractive for applications requiring repeated deformation, damage tolerance, and long-term structural stability, such as tissue engineering scaffolds and injectable biomaterials.

### 2.6. Antibacterial Activity of the COL/PAA/Fe Hydrogel

**Figure 6:**
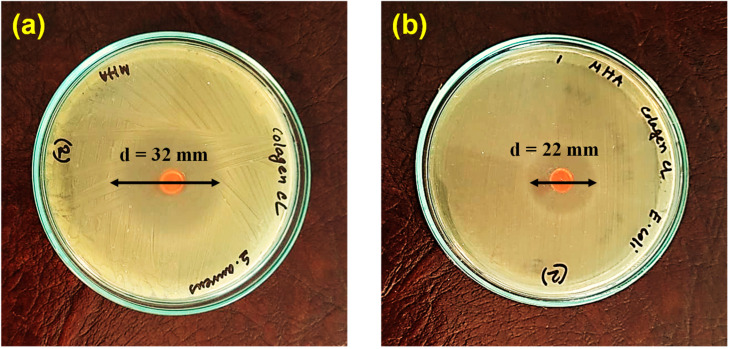
Antimicrobial activity of the COL/PAA/Fe hydrogels against (**a**) *S. aureus* and (**b**) *E. coli*.

Figure 6 shows the antibacterial performance of the COL/PAA/Fe hydrogels against G+ and G- bacteria. The synthesized hydrogel exhibited pronounced antibacterial activity against both strains, as evidenced by clear and well-defined zones of inhibition surrounding the samples. The diameters of the inhibition zones were measured to be 32 mm for *S. aureus* (Figure 6a) and 22 mm for *E. coli* (Figure 6b), indicating broad-spectrum antibacterial efficacy.

The antibacterial activity of the COL/PAA/Fe hydrogel is primarily attributed to the presence of Fe^3+^ ions within the polymer network. Positively charged Fe^3+^ ions can interact electrostatically with negatively charged bacterial cell membranes, leading to membrane disruption and loss of cellular integrity. Additionally, the Fe^3+^/Fe^2+^ redox couple can induce oxidative stress through Fenton-like reactions, further damaging bacterial proteins and genetic material [47,48]. The COL/PAA matrix, rich in ionizable –NH_2_, –OH, and –COO^−^ groups, may promote strong surface interactions with bacteria, facilitating Fe^3+^ binding and localized antimicrobial activity. Meanwhile, COL provides a biocompatible scaffold that supports uniform dispersion of active components without compromising antibacterial performance [49,50].

Notably, the hydrogel exhibited a larger inhibition zone against *S. aureus* (32 mm) than *E. coli* (22 mm). Positive control (medium + bacteria + antibiotic) and negative control (medium + bacteria) tests were also conducted (Figure S2). In case of positive control, Cefotaxime (30 µg) was used as an antibiotic, which showed inhibitory activity against both *S. aureus* and *E. coli*. The zone of inhibition included the antibiotic disc diameter, and it was 16.2 ± 0.2 and 22.2 ± 0.2 mm against *S. aureus* and *E. coli*, respectively. This can be attributed to the Gram-positive cell wall structure of *S. aureus* that permits greater Fe^3+^ penetration and enhanced generation of reactive oxygen species (ROS) via Fenton-like reactions, leading to more effective membrane damage and bacterial inactivation compared to Gram-negative *E. coli*, whose outer lipopolysaccharide layer provides additional protection [51]. Thus, these results confirm the strong antibacterial potential of the COL/PAA/Fe hydrogel for infection-resistant biomedical applications.

## 3. Conclusions

In summary, multifunctional COL/PAA/Fe hydrogels with tunable mechanical properties were successfully fabricated through APS-initiated polymerization and Fe^3+^-mediated coordination cross-linking. The synergistic interactions between COL and PAA, comprising extensive hydrogen bonding and dynamic Fe^3+^–carboxylate coordination, conferred the hydrogel network with improved mechanical strength, high elasticity, and autonomous self-healing properties. The hydrogels achieved the highest tensile and compressive strengths of ~0.176 MPa at 751% elongation and ~1.945 MPa at ~82% compressive strain, respectively. Moreover, a high IC of 4.11 S·m^−1^ was observed for the COL-5/PAA/Fe hydrogel, enabling reliable performance and structural integrity in flexible electronic applications. The resulting hydrogels demonstrated excellent structural integrity and durability, along with rapid autonomous self-healing behavior and effective antibacterial activity against both Gram-positive and Gram-negative bacteria. Owing to the biocompatible COL framework, coupled with the functional versatility of PAA and Fe^3+^ ions, the prepared hydrogels manifest strong potential for biomedical applications such as wound dressings, tissue engineering scaffolds, and injectable biomaterials. This strategy highlights the use of COL extracted from raw tannery trimmings as a sustainable precursor for hydrogel fabrication, while exploiting a redox-assisted polymerization and metal-ion coordination system to produce a mechanically stable and multifunctional material. The integration of sustainably sourced collagen into hydrogel systems presents a viable pathway toward eco-conscious biomaterial platforms that combine functional performance with environmental responsibility.

## 4. Materials and Methods

### 4.1. Materials

Trimmings of raw cowhide were supplied by the Savar Tannery Estate in Dhaka, Bangladesh. Methylene blue (MB), acrylic acid (AA), ammonium persulfate (APS) ((NH_4_)_2_S_2_O_8_), iron(III) chloride hexahydrate (FeCl_3_⋅6H_2_O), Nutrient Broth (NB), and Mueller−Hinton Agar were purchased from Darmstadt, Merck, Germany. All experiments were carried out using deionized (DI) water, and all analytical-grade reagents were directly used as supplied without any additional purification.

### 4.2. Extraction of COL from Raw Trimmings

**Figure 7:**
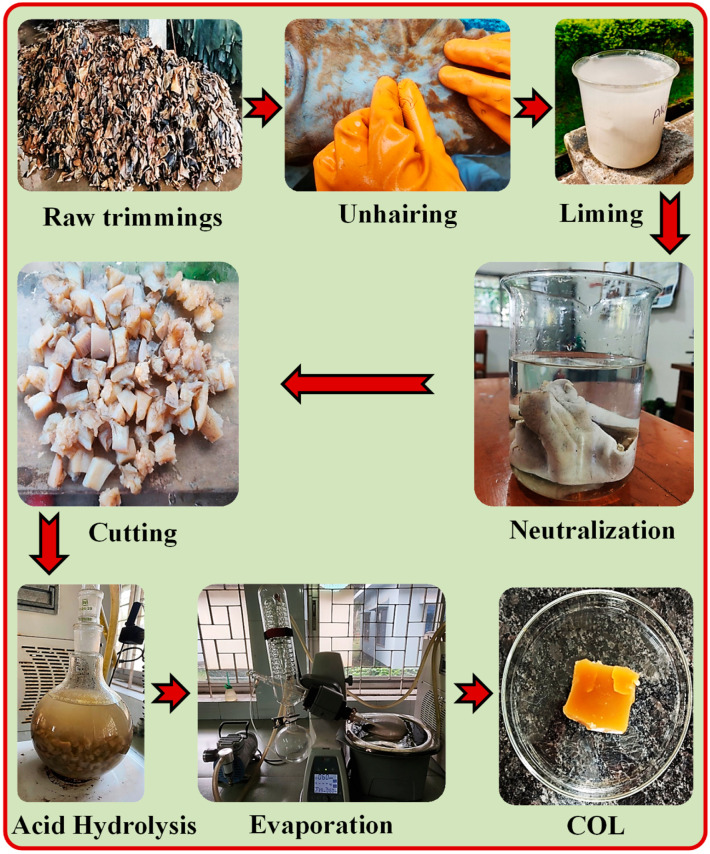
Stepwise illustration of the extraction of COL from raw trimmings.

COL was extracted from raw cowhide trimmings following an established method reported in the literature [52]. Initially, the raw trimmings were washed with plenty of tap water in order to get rid of dirt, salt, blood, dung, and other contaminants. After that, the trimmings were immersed in water until they obtained a moisture content of 60–65%. The dehairing and deliming processes were then carried out by chemical procedure. A total of 10% (% was calculated based on the weight of trimmings) of lime and 3% of Na_2_S were applied for 24 h for the unhairing process, and in the deliming process, 2% of NH_4_Cl was applied for 120 min. The delimed trimmings were then thoroughly rinsed with water until a neutral pH was achieved, and subsequently cut into 1 × 1 cm pieces. For COL extraction, 100 g of the prepared pieces were immersed in 300 mL of 0.5 M CH_3_COOH in a round-bottom flask. Later, the flask was placed on a magnetic hot plate stirrer and connected to the condenser. The mixture was heated to 60 °C for 18 h under continuous stirring at 600 rpm. Next, the hydrolysate was concentrated by evaporation until a semi-solid texture was attained which is called COL cake. The water content of the COL cake was 76% (*w*/*w*), and it was stored at −4 °C for further use. The extraction procedure of COL from raw tannery trimmings is illustrated in Figure 7.

COL was extracted from multiple batches of bovine trimming waste collected from the same tannery facility to ensure reproducibility. A consistent pretreatment and extraction protocol, including washing, acid swelling, purification, and drying, was applied to all batches. No noticeable differences in the physicochemical characteristics of the extracted COL were observed between batches.

### 4.3. Molecular Weight of COL

The molecular weight of the COL sample was determined by using GPC following reported literature methods [15]. The GPC analysis was conducted by applying a HPLC system (Agilent 1200, Santa Clara, CA, USA) with a TSK G2000 SWXL column (7.8 × 300 mm; particle size 7 μm) (TOSOH Co., Tokyo, Japan). Before starting the GPC test, 2 mL of buffer solution containing 0.1 M sodium phosphate buffer (PBS) and 0.1 M sodium sulfate (pH 6.7) was used to dissolve the CG sample. The mobile phase consisted of 0.1 M PBS with 0.1 M sodium sulfate (pH, 6.7) and flowed at a rate of 0.5 mL/min. The flow rate for the mobile phase was set to 0.5 mL/min. The absorbance of ultraviolet light was measured at 214 nm. Lab Solution software (Version 5) was utilized for the collection and analysis of data. Data analysis was carried out by applying one-way analysis of variance (ANOVA) through SPSS version 22 software for Windows (SPSS Inc., Chicago, IL, USA). The Mw and Mn of the COL were found to be 375,642 g/mol and 332,837 g/mol, respectively.

### 4.4. Preparation of the COL/PAA/Fe Hydrogel

**Figure 8:**
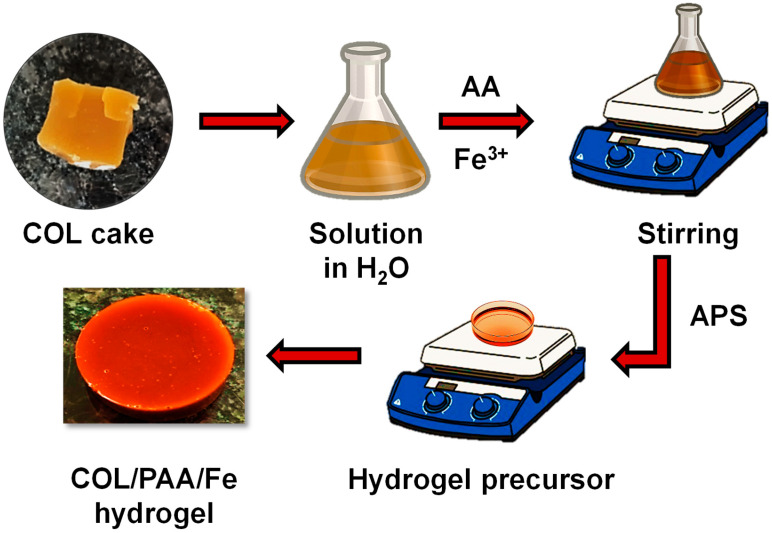
Schematics demonstrating the fabrication process of the COL/PAA/Fe hydrogel.

About 4.0 g of COL was initially dissolved in 3 mL of DI water in a conical flask and stirred until a homogeneous solution was obtained. After that, 2.7 mL of AA and 0.25 g of FeCl_3_·6H_2_O were added, and the resulting mixture was stirred on a hot plate at 45 °C. Subsequently, after the addition of approximately 0.2 g of APS powder, the hydrogel precursor was poured into a Petri dish, in which an exothermic polymerization reaction induced rapid gelation, forming the COL/PAA/Fe hydrogel within ~2 min. Figure 8 shows the schematic demonstration of the preparation process of the COL/PAA/Fe hydrogel.

Various quantities of COL (Table 1) and FeCl_3_·6H_2_O (Table 2) were utilized to synthesize different hydrogels for optimizing the hydrogel preparation process. In addition to the optimization of COL and Fe^3+^ dosages, other synthesis parameters, including the concentration of AA, the pH of the reaction medium, and reaction conditions, were carefully controlled and maintained constant to ensure reproducibility of the hydrogel formation.

### 4.5. Characterizations

FT-IR spectra of the prepared COL/PAA/Fe hydrogel and its precursors (COL and AA) were captured using a FT-IR spectrophotometer (Bruker, Fällanden, Zurich, Switzerland) equipped with a UATR Accessory in order to identify the presence of various functional groups. Each spectrum was recorded with 20 averaged scans at a resolution of 4 cm^−1^ over the 4000–600 cm^−1^ frequency range. The XPS analyses of COL, COL/Fe^3+^ chelated suspension, and COL/PAA/Fe hydrogel were studied on a Thermo Fisher Scientific K-Alpha (Thermo Scientific ESCALAB 250Xi, Waltham, MA 02451, USA) instrument.

### 4.6. Mechanical Behavior and IC Analysis

A universal testing machine (INSTRON^®^, Norwood, MA, USA) was used to evaluate the tensile strengths of the synthesized hydrogels. For this purpose, the hydrogel samples were prepared with a width of 11.2 mm and a thickness of 1.0 mm. Compressive strength measurements of the COL-5/PAA/Fe hydrogel were performed using a universal testing apparatus (Model: ZL-8001A, Zhongli Instrument Technology Co., Ltd., Dongguan, China). Prior to testing, the hydrogels were cut into cylindrical specimens with a height of 8.0 mm and a diameter of 25.0 mm to ensure uniformity.

IC of the prepared COL/PAA/Fe hydrogel was measured using an LCR meter (TH 2832, Changzhou Tonghui Electronic Co., Ltd., Changzhou, China). A sample of definite dimensions were cut from the intact hydrogel, maintaining an area (A) of 1 cm × 0.5 cm and a thickness (d) of 0.5 cm, which was then sandwiched between two parallel aluminum thin-film electrodes. The electrodes were connected to the LCR meter, and the resistivity (R) was measured under an applied frequency of 1 kHz and a voltage of 1 V. The IC (I) was finally determined by using Equation (1):

(1) $$I=\frac{d}{AR}$$

### 4.7. Self-Healing Properties

The self-healing performance of the COL/PAA/Fe hydrogel was evaluated using a visual macroscopic healing assay. Two hydrogel samples were prepared, one of which was stained with MB to facilitate visual observation, while the other remained unstained. Each hydrogel sample was bisected into two equal halves using a sharp blade. Immediately after cutting, the freshly exposed surfaces of the stained and unstained hydrogel pieces were brought into close contact and carefully aligned without applying external pressure. The assembled samples were kept undisturbed under ambient conditions for 5 min to allow autonomous self-healing. The restoration of structural integrity across the cut interface was visually examined. For quantitative measurement of self-healing performance of the hydrogel, the COL-5/PAA/Fe hydrogel was selected, and the abovementioned procedure was followed. After 5 min of resting, the tensile strength of the self-healed hydrogel was measured.

### 4.8. Antibacterial Activity

The antimicrobial properties of the prepared hydrogels were evaluated against both Gram-positive (G+) [*Staphylococcus aureus*] and Gram-negative (G-) [*Escherichia coli*] bacteria. The stock cultures were maintained on bacterial Mueller−Hinton agar medium through subculturing. Colonies from the freshly prepared plates were suspended in NB and incubated at 37 °C for 24 h.

## Figures and Tables

**Table 1:** Determination of the optimal COL content for COL/PAA/Fe hydrogel formation.

COL (g)	DI Water (mL)	AA (mL)	FeCl_3_·6H_2_O (g)	Hydrogel
0.0	3.0	2.7	0.25	No gel formation
1.0	3.0	2.7	0.00	No gel formation
1.0	3.0	2.7	0.00	No gel formation
1.0	3.0	2.7	0.25	COL-1/PAA/Fe
2.0	3.0	2.7	0.25	COL-2/PAA/Fe
3.0	3.0	2.7	0.25	COL-3/PAA/Fe
4.0	3.0	2.7	0.25	COL-4/PAA/Fe
5.0	3.0	2.7	0.25	COL-5/PAA/Fe
6.0	3.0	2.7	0.25	COL-6/PAA/Fe

**Table 2:** Optimization of the amount of FeCl_3_·6H_2_O based on the IC of COL/PAA/Fe hydrogel.

COL (g)	DI Water (mL)	AA (mL)	FeCl_3_·6H_2_O (g)	IC (S·m^−1^)
5.0	3.0	2.7	0.05	2.01
5.0	3.0	2.7	0.10	2.74
5.0	3.0	2.7	0.15	3.85
5.0	3.0	2.7	0.20	4.02
5.0	3.0	2.7	0.25	4.11
5.0	3.0	2.7	0.30	4.01

## Data Availability

All data presented or analyzed during this study are included in this article.

## Supplementary Materials

The following supporting information can be downloaded at: https://www.mdpi.com/article/10.3390/gels12040301/s1, Video S1: ionic conductivity of COL/PAA/Fe hydrogel; Video S2: elasticity and flexibility of COL/PAA/Fe hydrogel; Video S3: self-healing properties of COL/PAA/Fe hydrogel. Figure S1: Tensile stress and tensile strain curve of COL-5/PAA/Fe hydrogel and self-healed COL-5/PAA/Fe hydrogel; Figure S2: Antimicrobial activity of Cefotaxime (30 µg) against (a) *S. aureus* and (b) *E. coli*. and negative control against (c) *S. aureus* and (d) *E. coli.*

## Abbreviations

The following abbreviations are used in this manuscript:

AA	Acrylic acid
APS	Ammonium persulfate
COL	Collagen
FTIR	Fourier-transform infrared spectroscopy
GPC	Gel permeation chromatography
IC	Ionic conductivity
MB	Methylene blue
M_n_	Number average molecular weight
PAA	Poly(acrylic acid)
M_w_	Weight average molecular weight
XPS	X-ray photoelectron spectroscopy
UATR	Universal attenuated total reflectance
