# Supplementary material for: Efficient Recovery of Collagen from Tannery Waste Materials and Its Integration into Functional Hydrogel Systems
Source: Gels. 2026 Apr 1;12(4):301. doi: 10.3390/gels12040301 (PMC13115700; doi:10.3390/gels12040301)
Supplement: Supplementary file 1 [file gels-12-00301-s001.zip › Supporting information file.pdf]

Supporting Information for

Efficient Recovery of Collagen from Tannery Waste Materials and Its Integration into Functional Hydrogel Systems

Ilnaz Fargul Chowdhury[1], Akash Debnath[2], Shyama Prosad Moulick[3], Md. Ashraful Alam[4], S. M. Asaduzzaman Sujan[3], Md. Tushar Uddin[3], Md. Salim Khan[1], Ajoy Kanti Mondal[1,*]

[1] Institute of National Analytical Research and Service, Bangladesh Council of Scientific and Industrial Research, Dhanmondi, Dhaka 1205, Bangladesh

[2] Leather Research Institute, Bangladesh Council of Scientific and Industrial Research, Savar, Dhaka 1350, Bangladesh

[3] BCSIR Dhaka Laboratories, Bangladesh Council of Scientific and Industrial Research, Dhanmondi, Dhaka 1205, Bangladesh

[4] Institute of Energy Research and Development, Bangladesh Council of Scientific and Industrial Research, Dhanmondi, Dhaka 1205, Bangladesh

*Corresponding authors e-mail address: ajoymondal325@yahoo.com (Dr. A. K. Mondal).

1. Materials and Methods

1.1. Materials

Acetic acid (CH_3COOH) (Purity ≥ 99%), Calcium oxide (CaO), sodium sulfide (Na_2S), and ammonium chloride (NH_4Cl) were bought from Sigma-Aldrich, Germany. All the reagents were of analytical grade and they were used in this research without any further purification.

1.2. Mechanical Properties

Prior to measuring the tensile strengths of the prepared COL/PAA/Fe hydrogels, the gauge length between the two clamps of the instrument was 30 mm. The hydrogels were set in between the two clamps, and the tensile strengths were measured by applying the crosshead speed rate at 20 mm/min.

The hydrogel was placed in between the two jaws of the compression instrument in order to measure its compressive strength. The crosshead speed rate during compressive strength measurement was maintained at 10 mm/min. The tensile and compressive strengths (P) were calculated by following Eq. (S1):

$$P = \frac{F}{A} \quad \text{(S1)}$$

where F is the load and A is the original length or area of the hydrogel.

The toughness (Q) of the hydrogel was calculated by the area under the stress-strain curve from 0% strain to the cracking strain (ζ_β) by applying Eq. (S2), which is as follows:

$$Q = \int \sigma(\zeta) d\zeta \quad \text{(S2)}$$

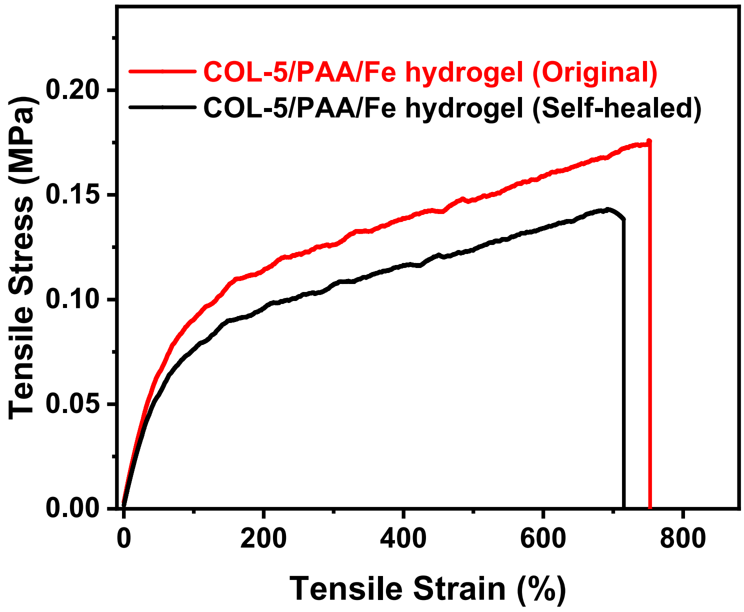

Figure S1. Tensile stress and tensile strain curve of COL-5/PAA/Fe hydrogel and self-healed COL-5/PAA/Fe hydrogel.

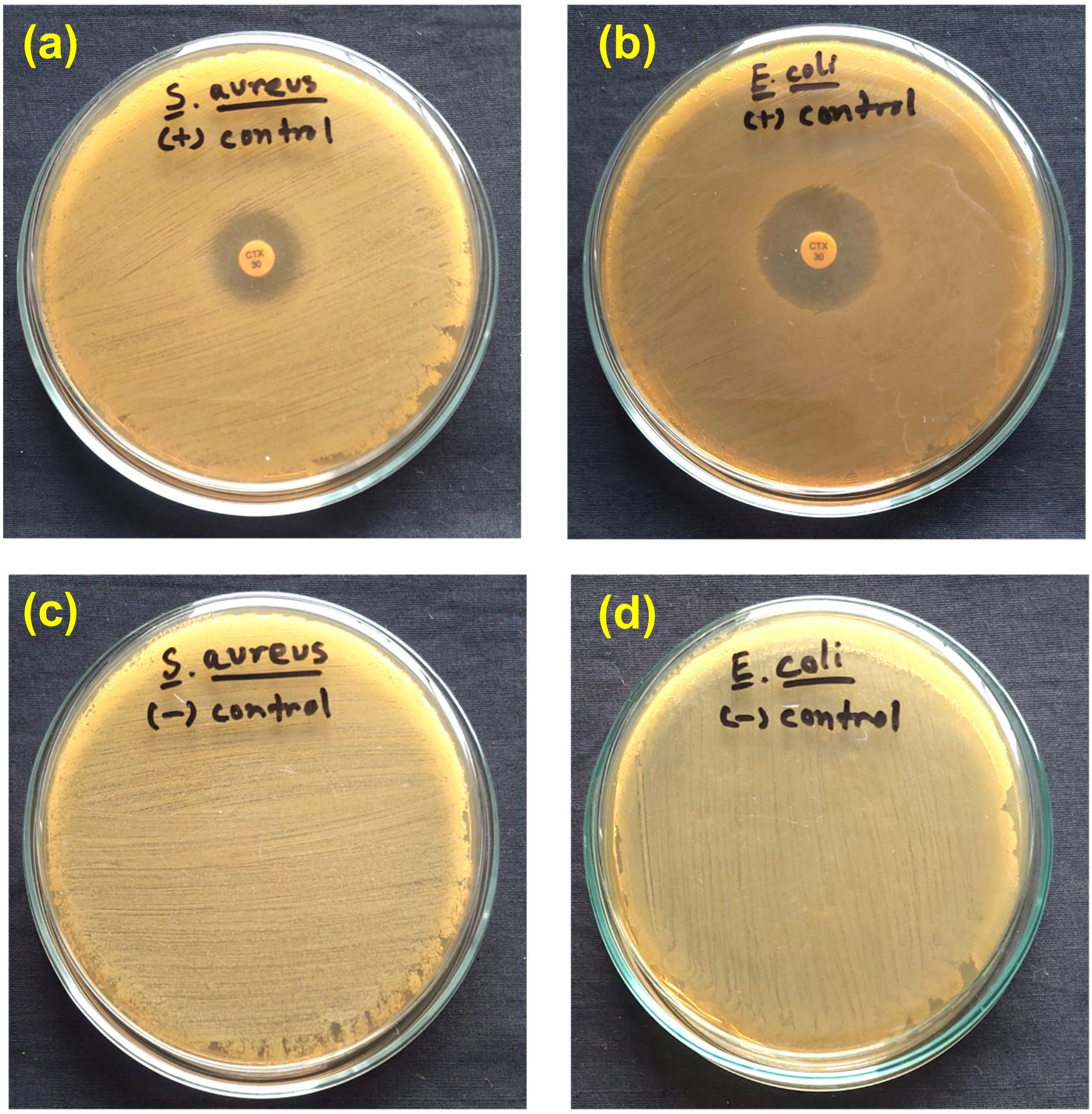

Figure S2. Antimicrobial activity of Cefotaxime (30 μg) against (a) S. aureus and (b) E. coli. and negative control against (c) *S. aureus* and (d) *E. coli.*
